# Development and Validation of a Score-Based Model for Estimating Esophageal Squamous Cell Carcinoma and Precancerous Lesions Risk in an Opportunistic Screening Population

**DOI:** 10.3390/cancers17132138

**Published:** 2025-06-25

**Authors:** Yan Bian, Ye Gao, Huishan Jiang, Qiuxin Li, Yuling Wang, Yanrong Zhang, Zhaoshen Li, Jinfang Xu, Luowei Wang

**Affiliations:** 1 Department of Gastroenterology, Changhai Hospital, Naval Medical University, Shanghai 200433, China; byan@smmu.edu.cn (Y.B.); gaoye@smmu.edu.cn (Y.G.); jianghuishan199711@163.com (H.J.); lqx3443353726@163.com (Q.L.); wangyl.2003@163.com (Y.W.); zhangyr977@163.com (Y.Z.); zhsl@vip.163.com (Z.L.); 2 Changhai Clinical Research Unit, Changhai Hospital, Naval Medical University, Shanghai 200433, China; 3 National Key Laboratory of Immunity and Inflammation, Naval Medical University, Shanghai 200433, China; 4 Department of Health Statistics, Naval Medical University, Shanghai 200433, China

**Keywords:** esophageal squamous cell carcinoma, precancerous lesions, risk stratification, opportunistic screening, individualized assessment

## Abstract

**Background:** Opportunistic screening is one major screening approach for esophageal squamous cell carcinoma (ESCC). We aimed to develop a score-based risk stratification model to assess the risk of ESCC and precancerous lesions in opportunistic screening and to validate it in an external population. **Methods:** The study was a secondary analysis of a published esophageal cancer screening trial. The trial was conducted in 39 secondary or tertiary hospitals in China, with 14,597 individuals including 71 high-grade intraepithelial neoplasia (HGIN) and 182 ESCC, enrolled for opportunistic screening. Additionally, questionnaires and endoscopy were performed. The primary outcome was histology-confirmed high-grade esophageal lesions, including HGIN and ESCC. The predictors were selected using univariable and multivariable logistic regression. Model performance was primarily measured with the area under the receiver operating characteristic curve (AUROC). **Results:** The score-based prediction model contained 8 variables on a 21-point scale. The model demonstrated an AUROC of 0.833 (95% CI, 0.803–0.862) and 0.828 (95% CI, 0.793–0.864) for detecting high-grade lesions in the training and validation cohorts, respectively. Using the cut-off score determined in the training cohort (≥9), the sensitivity reached 70.0% (95% CI, 50.6–85.3%), 81.3% (95% CI, 63.6–92.8%), and 81.1% (95% CI, 64.9–92.0%) in the validation cohort for detecting HGIN, early ESCC, and advanced ESCC, respectively, at a specificity of 76.4% (95%CI, 75.4–77.4%). The score-based model exhibited satisfactory calibration in the calibration plots. The model could result in 75.6% fewer individuals subjected to endoscopy. **Conclusions:** This score-based model demonstrated superior discrimination for esophageal high-grade lesions. It has the potential to inform referral decisions in an opportunistic screening setting.

## Simple Summary

Currently, there are no score-based models for estimating esophageal squamous cell carcinoma (ESCC) and precancerous lesions risk in an opportunistic population. The present study developed and validated a score-based risk prediction model for opportunistic screening for ESCC for the first time, comprising 8 variables on a 21-point scale. The model could detect 70.0%, 81.3%, and 81.1% of high-grade intraepithelial neoplasia, early ESCC, and advanced ESCC, respectively, with a specificity of 76.4%. Additionally, the score-based model could result in 75.6% fewer individuals subjected to endoscopy. The utilization of the score-based model enables risk stratification and individual self-assessment of ESCC during opportunistic screening.

## 1. Introduction

Esophageal cancer (EC) represented the 11th most prevalent and 7th most deadly malignant tumor globally, with an estimated 511,054 new cases and 445,391 deaths in 2022, respectively [1,2]. EC was classified into two main histological types: esophageal squamous cell carcinoma (ESCC) and esophageal adenocarcinoma (EAC) [3,4]. The risk factors, etiology, and distribution of ESCC and EAC differ significantly. EAC has been linked with Barrett’s esophagus, a history of gastroesophageal reflux disease, obesity, and tobacco smoking [5]. In contrast, ESCC has been associated with smoking and alcohol consumption. ESCC was predominantly concentrated in eastern and central Asia, sub-Saharan Africa, and some South American countries [6]. EAC, conversely, was mainly found in most countries in Europe, North America, and Oceania. ESCC represented over 85% of all cases [7]. It was estimated that approximately 50% of all EC cases occur in China. Furthermore, EC was the 7th most common cancer and the 5th most common cause of cancer-related mortality in China [8,9].

The absence of typical symptoms associated with early ESCC frequently resulted in the disease being diagnosed at an advanced stage, with a five-year survival rate of less than 30% [10,11]. Screening is an effective method for improving the early diagnosis of ESCC, which in turn enhances patients’ prognosis and quality of life [12,13]. Cancer screening programs could be categorized as either population-based or opportunistic screening. The implementation of mass population screening is challenging in areas with large populations due to the high cost and resource constraints involved. As a result, opportunistic screening is usually considered a viable alternative strategy [14,15]. Upper gastrointestinal endoscopy with targeted biopsy represented a validated approach to ESCC screening [16]. However, the high cost rendered it unsuitable for use in screening [12].

Risk stratification models could be used to assess each individual’s risk using relevant predictors so that only those at high risk are recommended for further endoscopy. This approach would allow the development of a cost-effective opportunistic screening strategy and holds promise in improving the sensitivity of endoscopic detection by alerting the endoscopists. However, the risk prediction model based on opportunistic screening populations remained inadequate. Liu et al. developed an ESCC predicting model for opportunistic screening constructed by logistic regression with five predictors [17]. The model proposed by Liu et al. employed alarm symptoms, such as dysphagia, as predictors, which may render the model more applicable to diagnosis than screening. Furthermore, the utilization of algorithms without a score assigned may impede the generalization of the model and patient self-assessment.

In this study, we developed and externally validated a score-based prediction model for estimating the risk of ESCC and precancerous lesions using data from a hospital-based opportunistic population in China. The score-based model facilitates the identification of individuals at high risk of ESCC, thereby enabling the implementation of further endoscopy in opportunistic screening.

## 2. Methods

### 2.1. Study Population

This study was a secondary analysis of a published nationwide, multicenter screening trial conducted in ESCC high-risk areas in China (Esophageal Cancer Screening Trial, ClinicalTrials.gov, NCT04609813). The participants in this study were recruited from 39 centers, all of which were either tertiary or secondary referral centers, between 1 January 2021 and 31 May 2022. The procedure of participant recruitment was based on the Esophageal Cancer Screening Trial, initiated by Shanghai Changhai Hospital [18]. The 21 centers situated in northern China were designated as the training cohort, whereas the 18 centers located in southern China were classified as the validation cohort. The geographic location of China is distinguished by the presence of the Qinling Mountain–Huaihe River Line, which serves to delineate the country’s southern and northern regions.

The participants in this study were consecutively recruited from outpatients at all study centers undergoing upper gastrointestinal endoscopy. The inclusion criteria were as follows: (1) age 40 to 75 years, (2) no history of esophageal neoplasia or cancer, (3) no alarming symptoms, including dysphagia, hematemesis, and melena, (4) written informed consent provided, and (5) an adequate upper gastrointestinal endoscopy undergone. A total of 14,597 participants were recruited, and the model was developed and tested by the pre-established training and validation cohorts.

The study was approved by the Shanghai Changhai Hospital Ethics Committee (No. CHEC2020-088) and was reviewed by all participating institutes. Written informed consent was obtained from all participants.

### 2.2. Study Procedures

As part of the Esophageal Cancer Screening Trial process, all enrolled participants completed a questionnaire and finally underwent upper gastrointestinal endoscopy.

#### 2.2.1. Questionnaire Survey

A structured online questionnaire was used in the present study. The questionnaire included baseline information (age, sex, residence, education level, body weight, and height), living styles (cigarette smoking [smoke more than one cigarette every day for more than one year; if yes, the number of cigarettes and duration were asked], alcohol drinking [alcohol drinking more than once every week for more than one year; if yes, the kind of wine, drinking frequency, and alcohol flushing were asked]), eating habits (hot food preference [hot food was defined as that had hot sensation in the mouth], pickled food preference [high frequency was defined as more than three times per week]), tooth loss, and family history of EC (first- or second-degree relatives).

#### 2.2.2. Upper Gastrointestinal Endoscopy Examination

All participants underwent upper gastrointestinal endoscopy. A white light view of the esophagus and stomach is taken, which is consistent with the approach used in the majority of endoscopy centers. Narrow-band imaging was requested to view the full length of the esophagus or Lugol’s chromoendoscopy for the examination of suspicious lesions. All suspicious lesions were subject to biopsy. The photographs or videos of upper gastrointestinal endoscopy were reviewed by two independent expert investigators. The biopsy specimens were evaluated by two experienced gastrointestinal pathologists following standard processing procedures. High-grade intraepithelial neoplasia (HGIN) and ESCC identified by biopsy were then subjected to further evaluation for the purpose of determining the standardized treatment. The pathology reports of endoscopic or surgical resection specimens were obtained for the purpose of further confirmation.

### 2.3. Outcomes

The primary outcome of this study was histology-confirmed, esophageal high-grade lesions, including squamous epithelial HGIN, early ESCC, and advanced ESCC. In this study, squamous epithelial HGIN was defined as the presence of dysplastic squamous epithelial cells that occupied over half of the whole epithelium. Early ESCC is confined to the mucosa, with no deeper involvement and no locoregional or distant spread [19]. Barrett’s esophagus, glandular epithelia HGIN, and EAC were not considered as the target outcomes for this study. The outcome measures included the area under the receiver operating characteristic curve (AUROC), sensitivity, specificity, accuracy rate, positive predictive value (PPV), negative predictive value (NPV), positive and negative likelihood ratio (LR), and the number needed to screen to detect one case of high-grade lesions (NNS).

### 2.4. Statistical Analysis

In the training cohort, univariable logistic regression was employed to ascertain risk factors for esophageal high-grade lesions, which were then utilized as candidate predictors for the construction of the model. Predictors with *p* value < 0.1 and odds ratio (OR) > 1.0 were subjected to multivariable logistic regression models. The exclusion of predictors from the multivariable logistic regression model was based on *p* values ≥ 0.1. This ensured that the final model included predictors with *p* value < 0.05 and OR > 1.0. Points were assigned by dividing the regression coefficients by the absolute value of the smallest coefficient of predictors and rounding up to the nearest 0.5. A score-based prediction model was constructed by aggregating the scores of each predictor for each participant.

Receiver operating characteristic curves (ROCs) were employed to evaluate the diagnostic efficacy of the model, and the score at the maximum of the Youden index was designated as the cut-off value. The score-based model and cut-off value were applied to the validation cohort for external validation and further analysis. The diagnostic performance of the score-based model was assessed by using AUROC, sensitivity, specificity, accuracy rate, PPV, NPV, positive LR, negative LR, and NNS. The calibration of the score-based model was assessed with calibration curves. Decision curve analysis (DCA) was used to report the net clinical benefit of the score-based model.

In this study, the comparison of the characteristics of the participants in the training and validation cohorts was conducted using the chi-squared test for categorical variables. All tests were two-sided and *p* values < 0.05 were considered to be significant. Analyses were performed with GraphPad Prism (version 9.0.0), MedCalc (version 20.022), and R software (version 4.2.2).

## 3. Results

### 3.1. Participant Characteristics

As shown in Table 1, a total of 7899 and 6698 participants were enrolled in the training and validation cohorts, respectively. In the training cohort, 153 patients were identified through histology as having esophageal high-grade lesions, comprising 41 HGIN, 27 early ESCC, and 86 advanced ESCC cases. The validation cohort comprised 98 esophageal high-grade lesions, including 30 HGIN, 32 early ESCC, and 37 advanced ESCC. Notably, one patient in each of the training and validation cohorts exhibited multiple malignant lesions, resulting in a discrepancy between the total number of lesions and the number of patients with high-grade lesions. Additionally, only BMI was balanced between the training and validation cohorts, while all other variables were significantly different. This indicated that there were significant differences in the characteristics of the opportunistic screening populations in southern and northern China, which could facilitate adequate validation of the model performance.

### 3.2. Development of the Scored-Based Prediction Model

To construct the model, 12 potential risk factors were selected as candidate predictors for the univariable logistic regression model (Table S1). The univariable logistic regression analysis revealed that the majority of the risk factors exhibited *p* values < 0.05, except alcohol flushing, hot food preference, and family history. Nevertheless, the univariable logistic regression *p* values for alcohol flushing and family history were less than 0.1, thus necessitating their inclusion in the multivariate logistic regression model.

The initial multivariable logistic regression model included 11 variables (Table S2). Of these, three variables (education level, alcohol drinking, and alcohol flushing) had *p* values ≥ 0.1, indicating that they were not statistically significant. Subsequently, the remaining eight variables (*p* < 0.1) were finally included in the multivariable logistic regression (Table 2). The results of the second multivariable logistic regression showed *p* values < 0.05 and ORs > 1.0 for all 8 variables.

The scores for each predictor are shown in Table 2, which constituted the score-based model for esophageal high-grade lesions as follows: age (4 for 50–59 years old; 6.5 for 60–69 years old; 9.5 for >69 years old), sex (2.5 for male), residence (2.5 for rural), BMI (1.5 for ≤22 kg/m^2^), cigarette smoking (1.5 for yes and smoking ≤ 30 pack-years, and 2 for yes and smoking > 30 pack-years), pickled food preference (1.5 for high), tooth loss (1.5 for >4), family history (1.5 for yes), with the total scores of each individual ranging from 0 to 21.

**Figure 1:**
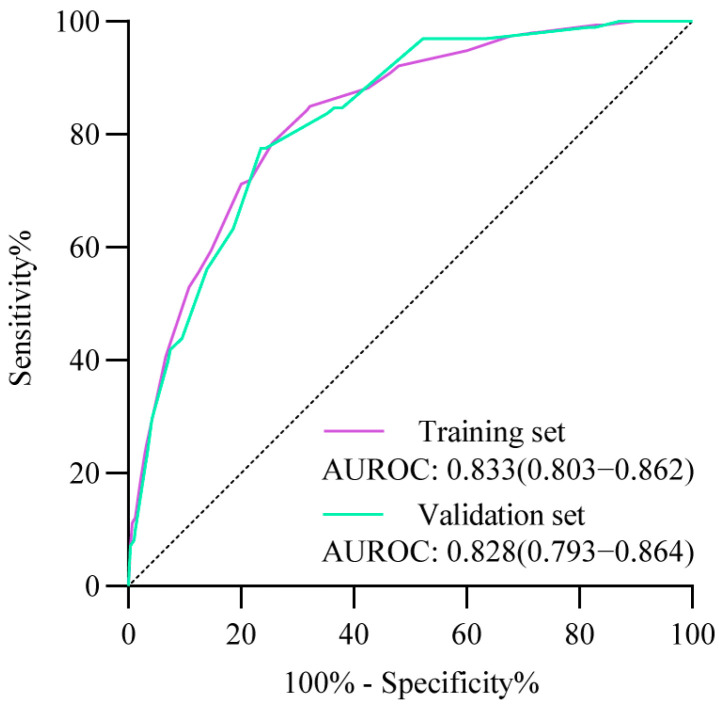
ROC for the score-based model in the training and validation cohorts. ROC, receiver operating characteristic curve; AUROC, area under the receiver operating characteristic curve.

The AUROC for the score-based model was 0.833 (95%CI, 0.803–0.862) in the training cohort (Figure 1). Table S3 shows the diagnostic performance of each score as a cut-off value in identifying individuals at high risk of high-grade lesions in the training cohort. As the score increases, the risk of high-grade lesions rises concomitantly with a decline in the proportion of recommendations for endoscopy and sensitivity. Conversely, specificity shows a gradual increase. A score of 9 at the maximum Youden Index (0.526) was selected as the cut-off value, with a sensitivity and specificity of 84.3% (95%CI, 77.6–89.7%) and 68.3% (95%CI, 67.3–69.4%), respectively. The sensitivity of this model for the detection of HGIN, early ESCC, and advanced ESCC was 82.9% (95%CI, 67.9–92.9%), 81.5% (95%CI, 61.9–93.7%), and 86.1% (95%CI, 76.9–92.6%), respectively (Table 3). Additionally, 32.7% of individuals were identified as high-risk individuals, and one case of high-grade lesions could be detected by performing 20 upper gastrointestinal endoscopies (Table 3).

### 3.3. External Validation of the Scored-Based Prediction Model

Furthermore, the scored-based model also showed excellent discriminative performance in the validation cohort. The AUROC for the score-based model was 0.828 (95%CI, 0.793–0.864) in the validation cohort (Figure 1). The overall sensitivity and specificity for the model were 77.6% (95%CI, 68.0–85.4%) and 76.4% (95%CI, 75.4–77.4%) at the cut-off score of 9, respectively (Table 3). The model exhibited a high capacity for distinguishing between various esophageal high-grade lesions, with a sensitivity of 70.0% (95%CI, 50.6–85.3%), 81.3% (95%CI, 63.6–92.8%), and 81.1% (95%CI, 64.9–92.0%) for HGIN, early ESCC, and advanced ESCC, respectively (Table 3). The model identified 24.4% of the individuals in the validation cohort as high-risk, indicating the need for further endoscopy (Table 3). Furthermore, the model demonstrated a significant reduction in the number of screening cases required to detect one case of high-grade lesions, from 68 to 21 (Table 3). The accuracy rate, PPV, NPV, positive LR, and negative LR are summarized in Table 3.

### 3.4. Model Calibration and Clinical Utility

**Figure 2:**
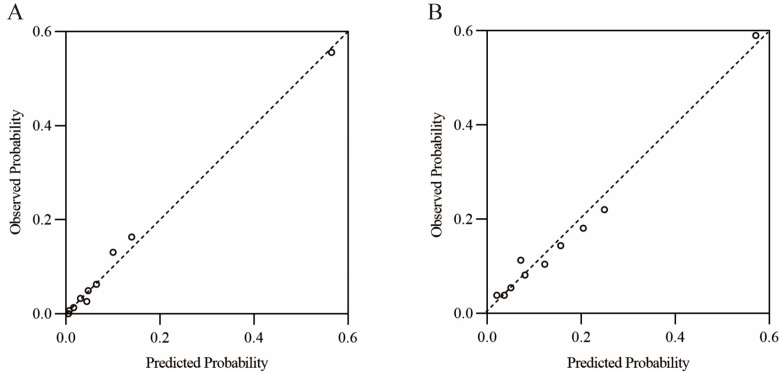
Hosmer–Lemeshow calibration plots for training cohort (**A**) and validation cohort (**B**).

The Hosmer–Lemeshow goodness-of-fit test and calibration plot analysis indicated that the score-based model demonstrated satisfactory calibration in both the training and validation cohorts (Table S4, Figure 2). Additionally, DCA demonstrated the net clinical benefit of utilizing the model in comparison to the alternative scenarios of all endoscopic screening and no endoscopic screening (Figure S1).

## 4. Discussion

In this study, we developed and externally validated a score-based model to identify the high-risk individuals for esophageal high-grade lesions in Chinese opportunistic screening. The model comprises eight predictors on a 21-point scale, with a score of 9 serving as the cut-off value, indicating high-risk individuals (Figure S2). The model could detect 70.0%, 81.3%, and 81.1% of HGIN, early ESCC, and advanced ESCC, respectively, with a specificity of 76.4%. Additionally, the score-based model could result in 75.6% fewer individuals subjected to endoscopy. Previous studies of ESCC risk stratification models were usually based on a general population, overlooking the development of risk stratification models appropriate for an opportunistic population [20,21,22,23]. The risk stratification model developed by Liu et al. was based on an opportunistic population. However, this model was calculated by a logistic regression algorithm, which is not easily and quickly calculable by users. Additionally, Liu et al.’s model incorporated alarm symptoms as predictors, which may impede the model’s capacity to detect early lesions. To our knowledge, the model in this study was the first score-based model constructed for an opportunistic population, exhibiting superior discriminatory capacity for different grades of esophageal malignancy lesions. In this study, particular emphasis was placed on the identification of early lesions, with a specific focus on lesions that could be endoscopically resected in a curative manner. This approach has the potential to enhance the long-term prognosis and quality of survival for patients. Additionally, the score-based model demonstrated robust discriminative efficacy on both the training cohort and the external validation cohort, comprising a significantly heterogeneous population. This indicated that the model may have general clinical applications. The model could facilitate the assessment of the patient’s risk of ESCC during opportunistic screening, thereby informing endoscopic referral decisions. Furthermore, it could alert endoscopists to individuals at high risk of ESCC.

It is recommended that the range of ESCC screening modalities be expanded to facilitate more effective early detection and treatment. Currently, organized population screening has yielded favorable outcomes in several high-risk regions within China [23,24]. However, the potential for nationwide expansion is constrained by the vast population size and the relatively limited availability of medical resources. It can be reasonably proposed that the introduction of opportunistic screening can serve to complement the existing screening system for ESCC. Additionally, the target population for opportunistic screening is typically characterized by a higher level of compliance. Furthermore, the score-based risk stratification model could assist physicians from different specialties in making standardized referral decisions and facilitate the implementation of opportunistic screening in a manner that is both straightforward and efficient. In addition, the utilization of score-based scales for patient self-assessment has the potential to enhance patient motivation to engage in screening activities. Compared to previous studies [17,25], the opportunistic screening population included in this study was larger, covered a wider geographic area, and included more precancerous lesions and early ESCC. This could enhance the extrapolation of the study and the potential for clinical applications.

The majority of the eight predictors included in the model constructed for this study have been previously identified as risk factors for ESCC [21]. Furthermore, a variety of methods were employed to guarantee the precision of data collection, including the precise delineation of risk factors, the provision of training for researchers, and other related strategies. Nevertheless, there was a possibility of inaccuracies in the information provided on specific factors, including the quantification of alcohol intake and the temperature of the food. The predictors in the final model are characterized by easy-to-collect information and high accuracy, thus ensuring a high degree of reliability. Predictors such as cigarette smoking, pickled food preference, and family history have been used in previous predictive models for ESCC and are proven risk factors for ESCC [21]. Alcohol drinking, alcohol flushing, hot food preference, and education level were excluded from the final model because they were not significant in the multivariable logistic regression model. In addition, residence was included as a predictor in this study, a factor that has rarely been used in previous studies [20,22,23]. This may be because the populations included in the previous studies were all from high-risk rural areas. Furthermore, this study was the first to include tooth loss as a predictor. Some studies have demonstrated that tooth loss is an indicator of oral microbial dysbiosis, which can subsequently lead to an increased risk of developing ESCC [26,27,28]. In this study, individuals presenting with alarming symptoms were excluded, as these individuals may already have ESCC and thus require endoscopy, which would diminish the potential benefit of early diagnosis and screening from the prediction model [29].

By reviewing previous studies, risk stratification models that use only epidemiological factors to achieve similar diagnostic efficacy have been shown to be sufficiently superior [20,21,22,23,24,25]. The objective of this study is to devise a risk stratification scale that is both rapid and straightforward to utilize, with the aim of facilitating the rapid referral of opportunistic populations. In the pursuit of enhancing the diagnostic efficacy of the model, there is a potential necessity to consider incorporating diagnostic molecular markers or advanced imaging techniques in subsequent studies [12,13,30,31]. Furthermore, the validation of the model on more diverse or population-based cohorts is necessary. In subsequent studies, we will collaborate with villages or communities to invite populations to participate in an ESCC screening study with endoscopy by message, and to send them an invitation to self-assess using the scale in this study. Therefore, the scale’s diagnostic efficacy in a general population could be assessed.

Furthermore, the cut-off score in this study could be adaptable, according to clinically applicable scenarios. For instance, the cut-off scores are appropriately adjusted downward in high-risk and medically adequate areas to increase sensitivity. In low-risk areas, the cut-off scores can be appropriately adjusted upwards to ensure a lower false-positive rate. However, the cut-off scores’ adjustment strategy must be further explored and validated in future studies. Nevertheless, irrespective of the cut-off scores selected, it is inevitable that some of the early lesions will be overlooked. Consequently, individuals considered to be at high risk, who are capable of doing so, should be advised to undergo upper gastrointestinal endoscopy at the appropriate time [32].

The present study also has several potential limitations. Firstly, despite the large sample size and nationwide recruitment from 39 centers, it remains challenging to provide a comprehensive representation of the opportunistic population. Secondly, there is a possibility of recall and self-reporting bias in questionnaires, despite the study having been clearly defined and standardized training being conducted. Furthermore, the study population was not followed up, thus precluding the determination of the incidence of ESCC in high-scoring individuals in future years.

## 5. Conclusions

We have developed a score-based risk prediction model for ESCC and precancerous lesions based on eight epidemiological factors in the opportunistic population. The model demonstrated a high degree of accuracy in its predictive capabilities, and its performance has been validated in an independent population. The study yielded an accessible tool for clinical practice that could assist physicians of different specialties in making referral decisions and self-assessment for the risk of ESCC without additional financial burden.

## Figures and Tables

**Table 1:** Baseline characteristics of the study population.

Variable	Training Cohort (*n* = 7899)	Validation Cohort (*n* = 6698)	*p* Value
Age, years			<0.001
40–49	1883 (23.8%)	1675 (25.0%)	
50–59	3295 (41.7%)	2912 (43.5%)	
60–69	2153 (27.3%)	1630 (24.3%)	
>69	568 (7.2%)	481 (7.2%)	
Sex			<0.001
Female	4058 (51.4%)	3626 (54.1%)	
Male	3841 (48.6%)	3072 (45.9%)	
Residence			<0.001
Urban	5299 (67.1%)	6152 (91.8%)	
Rural	2600 (32.9%)	546 (8.2%)	
Education level			<0.001
Primary school or below	4739 (60.0%)	5542 (82.7%)	
Middle school or above	3160 (40.0%)	1156 (17.3%)	
BMI, kg/m^2^			0.735
≤22	2094 (26.5%)	1793 (26.8%)	
>22	5805 (73.5%)	4905 (73.2%)	
Cigarette smoking			<0.001
No	6386 (80.8%)	5535 (82.6%)	
Yes, pack-years			
≤30	1107 (14.0%)	971 (14.5%)	
>30	406 (5.1%)	192 (2.9%)	
Alcohol drinking			<0.001
Yes	2230 (28.2%)	1310 (19.6%)	
No	5669 (71.8%)	5388 (80.4%)	
Alcohol flushing			<0.001
Yes	268 (3.4%)	96 (1.4%)	
No	7631 (96.6%)	6602 (98.6%)	
Hot food preference			<0.001
Yes	3915 (49.6%)	2889 (43.1%)	
No	3984 (50.4%)	3809 (56.9%)	
Pickled food preference			<0.001
High	938 (11.9%)	198 (3.0%)	
Low	6961 (88.1%)	6500 (97.0%)	
Tooth loss			<0.001
≤4	6734 (85.3%)	5961 (89.0%)	
>4	1165 (14.7%)	737 (11.0%)	
Family history			<0.001
Yes	1250 (15.8%)	586 (8.7%)	
No	6649 (84.2%)	6112 (91.3%)	
Detected lesions			0.006
HGIN	41 (0.5%)	30 (0.4%)	
early ESCC	27 (0.3%)	32 (0.5%)	
advanced ESCC	86 (1.1%)	37 (0.6%)	
Patients with high-grade lesions	153 (1.9%)	98 (1.5%)	

Note: Because one patient had multiple lesions in the training and validation cohorts, ESCC or HGIN do not add up for patients with high-grade lesions. Abbreviations: HGIN, high-grade intraepithelial neoplasia; ESCC, esophageal squamous cell carcinoma; BMI, body mass index.

**Table 2:** Risk factors associated with high-grade lesions in the final multivariable logistic model and the assigned scores.

Variable	Regression Coefficient (95%CI)	Adjusted OR (95%CI)	*p* Value	Assigned Scores
Age, years				
40–49	Reference			0
50–59	1.360 (0.425–2.577)	3.895 (1.530–13.152)	0.011	4
60–69	2.396 (1.498–3.594)	10.974 (4.471–36.390)	<0.001	6.5
>69	3.320 (2.388–4.537)	27.656 (10.894–93.415)	<0.001	9.5
Sex				
Female	Reference			0
Male	0.850 (0.443–1.269)	2.340 (1.558–3.559)	<0.001	2.5
Residence				
Urban	Reference			0
Rural	0.358 (0.016–0.699)	1.431 (1.016–2.012)	0.040	1
BMI, kg/m^2^				
>22	Reference			0
≤22	0.549 (0.206–0.886)	1.731 (1.229–2.425)	0.002	1.5
Cigarette smoking				
No	Reference			0
Yes, pack-years				
≤30	0.462 (0.014–0.896)	1.587 (1.014–2.449)	0.039	1.5
>30	0.703 (0.206–1.180)	2.019 (1.229–3.256)	0.005	2
Pickled food preference				
Low	Reference			0
High	0.497 (0.062–0.905)	1.643 (1.064–2.472)	0.021	1.5
Tooth loss				
≤4	Reference			0
>4	0.501 (0.133–0.862)	1.651 (1.142–2.368)	0.007	1.5
Family history				
No	Reference			0
Yes	0.475 (0.051–0.874)	1.609 (1.052–2.395)	0.023	1.5

Abbreviations: CI, confidence interval; OR, odds ratio; BMI, body mass index.

**Table 3:** The predictive performance of the ESCC risk stratification scale at a cut-off score of 9.

Variable	Training Cohort	Validation Cohort
High-risk individuals (n, %)	2606 (32.7)	1632 (24.4)
True high-grade lesions cases (n)	129	76
Sensitivity (%, 95%CI)		
High-grade lesions cases	84.3 (77.6–89.7)	77.6 (68.0–85.4)
HGIN	82.9 (67.9–92.9)	70.0 (50.6–85.3)
Early ESCC	81.5 (61.9–93.7)	81.3 (63.6–92.8)
Advanced ESCC	86.1 (76.9–92.6)	81.1 (64.9–92.0)
Specificity (%, 95%CI)	68.3 (67.3–69.4)	76.4 (75.4–77.4)
Accuracy rate (%, 95%CI)	68.6 (67.6–69.7)	76.4 (75.4–77.5)
PPV (%, 95%CI)	5.0 (4.7–5.4)	4.7 (4.2–5.2)
NPV (%, 95%CI)	99.5 (99.3–99.7)	99.6 (99.4–99.7)
Positive LR (95%CI)	2.662 (2.468–2.872)	3.289 (2.932–3.690)
Negative LR (95%CI)	0.230 (0.159–0.332)	0.294 (0.203–0.425)
NNS	20	21

Abbreviations: HGIN, high-grade intraepithelial neoplasia; ESCC, esophageal squamous cell carcinoma; PPV, positive predictive value; NPV, negative predictive value; LR, likelihood rate; NNS, number needed to screen to detect one case of HGL; CI, confidence interval.

## Data Availability

Data will not be made publicly available; written requests to share clinical data from this work will require approval from the institutional review boards and legal departments of participating sites.

## Supplementary Materials

The following supporting information can be downloaded at: https://www.mdpi.com/article/10.3390/cancers17132138/s1. Figure S1: Decision curve analysis for the training cohort (A) and validation cohort (B); Figure S2: The prediction scale for estimating esophageal squamous cell carcinoma and precancerous lesions risk in opportunistic screening population; Table S1: Risk factors associated with high-grade lesions in the univariable logistic regression; Table S2: Risk factors associated with high-grade lesions in the intermediate multivariable logistic model; Table S3: Performance of the risk score-based model for high-grade lesions with different score cut-off values in the training cohort; Table S4: Statistics of the Hosmer–Lemeshow goodness-of-fit tests.
