# Supplementary material for: Development and Validation of a Score-Based Model for Estimating Esophageal Squamous Cell Carcinoma and Precancerous Lesions Risk in an Opportunistic Screening Population
Source: Cancers (Basel). 2025 Jun 25;17(13):2138. doi: 10.3390/cancers17132138 (PMC12249110; doi:10.3390/cancers17132138)
Supplement: Supplementary file 1 [file cancers-17-02138-s001.zip › cancers-3681475-supplementary.pdf]

Supplementary Materials

Contents

Figure S1. Decision curve analysis for the training cohort and validation cohort.
Figure S2. The prediction scale for estimating esophageal squamous cell carcinoma and precancerous lesions risk in opportunistic screening population.
Table S1. Risk factors associated with high-grade lesions in the univariable logistic regression.
Table S2. Risk factors associated with high-grade lesions in the intermediate multivariable logistic model.
Table S3. Performance of the risk score-based model for high-grade lesions with different score cut-off values in the training cohort.
Table S4. Statistics of the Hosmer Lemeshow good-of-fit tests.

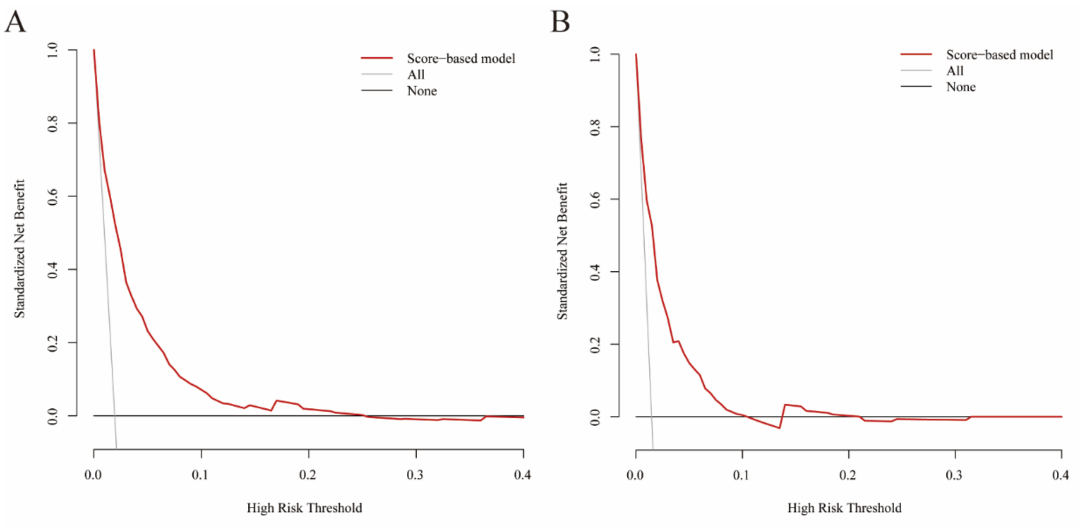

Figure S1. Decision curve analysis for the training cohort(A) and validation cohort(B).

**Variable**	**Score criteria**	
**Age(year)**	☐ 0	40-49
	☐ 4	50-59
	☐ 6.5	60-69
	☐ 9.5	＞69
**Sex**	☐ 0	Female
	☐ 2.5	Male
**Residence**	☐ 0	Urban
	☐ 1	Rural
**BMI(kg/m^2)**	☐ 0	＞22
	☐ 1.5	≤22
**Cigarette smoking (pack-years)**	☐ 0	No
	☐ 1.5	≤30
	☐ 2	＞30
**Pickled food preference**	☐ 0	Low(≤3 times per week)
	☐ 1.5	High(＞3 times per week)
**Tooth loss(n)**	☐ 0	≤4
	☐ 1.5	＞4
**Family history**	☐ 0	No
	☐ 1.5	Yes(first- or second-degree relatives with esophgeal cancer)
**Total score**		

Figure S2. The prediction scale for estimating esophageal squamous cell carcinoma and precancerous lesions risk in opportunistic screening population.

Table S1. Risk factors associated with high-grade lesions in the univariable logistic regression.

Variable	Regression coefficient(95%CI)	OR (95%CI)	P value
Age, years			
40-49	Reference		
50-59	1.462(0.531-2.678)	4.316(1.701-14.551)	0.006
60-69	2.759(1.876-3.950)	15.786(6.529-51.926)	<0.001
>69	3.792(2.890-4.993)	44.35(17.993-147.359)	<0.001
Sex			
Female	Reference		
Male	1.148(0.791-1.526)	3.152(2.206-4.600)	<0.001
Residence			
Urban	Reference		
Rural	0.738(0.417-1.060)	2.092(1.518-2.885)	<0.001
Education level			
Middle school or above	Reference		
Primary school or below	0.479(0.135-0.839)	1.614(1.145-2.313)	0.007
BMI, kg/m^2			
> 22	Reference		
≤22	0.733(0.405-1.056)	2.081(1.500-2.874)	<0.001
Cigarette smoking			
No	Reference		
Yes, pack-years			
≤30	0.841(0.432-1.228)	2.318(1.540-3.415)	<0.001
> 30	1.716(1.274-2.131)	5.563(3.577-8.427)	<0.001
Alcohol drinking			
No	Reference		
Yes	0.566(0.178-0.931)	1.761(1.195-2.536)	0.003
Alcohol flushing			
No	Reference		
Yes	0.592(-0.167-1.219)	1.807(0.847-3.384)	0.090
Hot food preference			
No	Reference		
Yes	-0.156(-0.479-0.165)	0.856(0.619-1.179)	0.341
Pickled food preference			
Low	Reference		
High	0.692(0.280-1.074)	1.997(1.323-2.928)	<0.001
Tooth loss			
≤4	Reference		
>4	1.326(0.989-1.654)	3.764(2.689-5.228)	<0.001
Family history			
No	Reference		
Yes	0.349(-0.061-0.730)	1.417(0.941-2.075)	0.083

Abbreviations: CI, confidence interval; OR, odds ratio; BMI, body mass index.

Table S2. Risk factors associated with high-grade lesions in the intermediate multivariable logistic model.

Variable	Regression coefficient(95%CI)	Adjusted OR (95%CI)	P value
Age, years			
40-49	Reference		
50-59	1.342(0.407-2.559)	3.826(1.502-12.924)	0.012
60-69	2.358(1.458-3.558)	10.572(4.299-35.097)	<0.001
>69	3.265(2.329-4.485)	26.179(10.269-88.645)	<0.001
Sex			
Female	Reference		
Male	0.879(0.467-1.304)	2.409(1.594-3.683)	<0.001
Residence			
Urban	Reference		
Rural	0.340(-0.004-0.683)	1.405(0.996-1.979)	0.052
Education level			
Middle school or above	Reference		
Primary school or below	0.237(-0.128-0.616)	1.268(0.880-1.852)	0.210
BMI, kg/m^2			
> 22	Reference		
≤22	0.548(0.206-0.886)	1.730(1.228-2.425)	0.002
Cigarette smoking			
No	Reference		
Yes, pack-years			
≤30	0.483(0.007-0.943)	1.621(1.007-2.567)	0.043
> 30	0.715(0.188-1.223)	2.044(1.207-3.396)	0.007
Alcohol drinking			
No	Reference		
Yes	-0.037(-0.538-0.442)	0.964(0.584-1.556)	0.883
Alcohol flushing			
No	Reference		
Yes	0.015(-0.834-0.776)	1.016(0.434-2.174)	0.97
Pickled food preference			
Low	Reference		
High	0.533(0.090-0.950)	1.704(1.094-2.586)	0.015
Tooth loss			
≤4	Reference		
>4	0.484(0.115-0.846)	1.623(1.122-2.331)	0.009
Family history			
No	Reference		
Yes	0.478(0.053-0.877)	1.613(1.055-2.403)	0.022

Abbreviations: CI, confidence interval; OR, odds ratio; BMI, body mass index.

Table S3. Performance of the risk score-based model for high-grade lesions with different score cut-off values in the training cohort.

Score	High-risk individuals (n, %)	True high-grade lesions (n)	True non high-grade lesions (n)	Sensitivity (%)	Specificity (%)	Youden index	Accuracy rate (%)	NNS
1	7530(95.3)	153	369	100.0	4.8	0.048	6.6	49
2	7119(90.1)	153	780	100.0	10.1	0.101	11.8	47
3	6696(84.8)	152	1202	99.4	15.5	0.149	17.1	44
4	6586(83.4)	152	1312	99.4	16.9	0.163	18.5	43
5	5737(72.6)	150	2159	98.0	27.9	0.259	29.2	38
6	4790(60.6)	145	3101	94.8	40.0	0.348	41.1	33
7	3858(48.8)	141	4029	92.2	52.0	0.442	52.8	27
8	3431(43.4)	135	4450	88.2	57.5	0.457	58.0	25
9	2582(32.7)	129	5293	84.3	68.3	0.526	68.6	20
10	1783(22.6)	110	6073	71.9	78.4	0.503	78.3	16
11	1228(15.5)	91	6609	59.5	85.3	0.448	84.8	13
12	920(11.6)	81	6907	52.9	89.2	0.421	88.5	11
13	576(7.3)	62	7232	40.5	93.4	0.339	92.3	9
14	287(3.6)	38	7497	24.8	96.8	0.216	95.4	8
15	195(2.5)	28	7579	18.3	97.8	0.161	96.3	7
16	98(1.2)	18	7666	11.8	99.0	0.107	97.3	5
17	34(0.4)	9	7721	5.9	99.7	0.056	97.9	4
18	21(0.3)	5	7730	3.3	99.8	0.031	97.9	4
19	9(0.1)	2	7739	1.3	99.9	0.012	98.0	5

Note: There were no high-grade lesions cases in the score of 20 or 21; therefore, we do not display the corresponding statistics of these two cut-off values.

Abbreviations: NNS, number needed to screen to detect one case of high-grade lesions.

Table S4. Statistics of the Hosmer Lemeshow good-of-fit tests.

Group	χ^2	P value
Training cohort	7.396	0.495
Validation cohort	4.359	0.823
